# Electroactive Poly(amic acid) Films Grafted with Pendant Aniline Tetramer for Hydrogen Sulfide Gas Sensing Applications

**DOI:** 10.3390/polym17141915

**Published:** 2025-07-11

**Authors:** Kun-Hao Luo, Yun-Ting Chen, Hsuan-Yu Wu, Zong-Kai Ni, Jui-Ming Yeh

**Affiliations:** Department of Chemistry, Chung Yuan Christian University, Chung Li District‚ Taoyuan City 32023, Taiwan

**Keywords:** pendant aniline tetramer, poly(amic acid), electroactive polymer, hydrogen sulfide, gas sensing

## Abstract

Hydrogen sulfide (H_2_S) is a highly toxic and corrosive gas generated in numerous industrial and environmental processes; rapid, sensitive detection at low ppm levels is therefore crucial for ensuring occupational safety and protecting public health. This work explores the effect of grafting various loadings of pendant aniline tetramer pendants (PEDA) onto electroactive poly(amic acid) (EPAA) films and evaluates their performance as H_2_S gas sensors. Comprehensive characterization including ion trap mass spectrometry (Ion trap MS), Fourier-transform infrared spectroscopy (FTIR), cyclic voltammetry (CV), and four-probe conductivity measurements, confirmed successful PEDA incorporation and revealed enhanced electrical conductivity with increasing PEDA content. Gas sensing tests revealed that EPAA3 (3 wt% PEDA) achieved the best overall performance toward 10 ppm H_2_S, producing a 591% response with a rapid 108 s response time. Selectivity studies showed that the response of EPAA3 to H_2_S exceeded those for SO_2_, NO_2_, NH_3_, and CO by factors of five to twelve, underscoring its excellent discrimination against common interferents. Repeatability tests over five successive cycles gave a relative standard deviation of just 7.4% for EPAA3, and long-term stability measurements over 16 days in ambient air demonstrated that EPAA3 retained over 80%. These findings establish that PEDA-grafted PAA films combine the processability of poly(amic acid) with the sharp, reversible redox behavior of pendant aniline tetramers, delivering reproducible, selective, and stable H_2_S sensing. EPAA3, in particular, represents a balanced composition that maximizes sensitivity and durability, offering a promising platform for practical environmental monitoring and industrial safety applications.

## 1. Introduction

Hydrogen sulfide (H_2_S) is a highly toxic, colorless, flammable gas produced by both natural decay and many industrial processes (e.g., oil refining, wastewater treatment, pulp/paper mills). It is recognized as one of the most dangerous workplace gases [1]. Even low-ppm exposures can have serious effects: for example, ~2 ppm can induce nausea, ~20 ppm causes loss of smell, ~100 ppm causes severe respiratory irritation, and concentrations above ~250 ppm can be fatal [2]. Consequently, stringent exposure limits have been set (from 1 ppb to 100 ppm, depending on duration) [3]. Continuous, real-time detection of H_2_S at sub-ppm levels is therefore critically important for environmental monitoring and occupational safety.

Semiconductor-based chemiresistors (e.g., ZnO nanowires, SnO_2_ quantum dots) and hybrid quantum-dot sensors have demonstrated excellent sensitivity and fast response to H_2_S, but often require high operating temperatures, complex nanofabrication, or suffer from baseline drift under humid conditions [4,5]. For example, SnO_2_ NFs/rGO composites yield ppb-level detection yet demand > 200 °C for reliable operation, limiting their practical deployment in portable or low-power systems [6]. Conducting polymers (CPs), such as polyaniline (PANI) and polypyrrole, offer an attractive alternative [7]: they operate at room temperature, are solution-processable, and feature tunable redox activity for gas interactions [8,9]. In particular, PANI has been extensively studied for H_2_S sensing [8]. Park et al. presented a room temperature H_2_S gas sensor based on electrospun polyaniline–polyethylene oxide (PANI–PEO) nanofibers doped with camphorsulfonic acid (HCSA); it operates by H_2_S further doping the PANI chains, as this process produces a dramatic increase in polymer conductivity [10]. Chang et al. delivered a H_2_S gas sensor based on an in situ polymerized SnO_2_/rGO/PANI ternary nanocomposite which operates at room temperature and exhibits high sensitivity, achieving a response of 23.9 towards 200 ppb H_2_S and a detection limit of 50 ppb [11]. Such composite approaches exploit PANI’s facile doping mechanism to amplify the H_2_S response.

Despite these advantages, conventional CP sensors often suffer from key limitations. Intrinsic CP films (without inorganic additives) tend to exhibit poor selectivity and limited stability when used for gas sensing [12]. For instance, proton-doped PANI (emeraldine salt) is known to lose its dopant ions over time (ions can migrate or volatilize), leading to baseline drift and reduced reproducibility [8]. Moreover, PANI itself is notoriously difficult to process: its poor solubility and tendency to aggregate make it hard to form uniform, defect-free films [13]. These issues (unstable doping, film inhomogeneity, cross-sensitivity to humidity and other gases) have motivated the search for new polymer architectures with better controlled conductivity and film-forming properties.

To overcome these challenges, recent research has explored the incorporation of electroactive aniline oligomers—such as dimers, trimers, and tetramers—into polymer matrices. These oligomers exhibit well-defined redox transitions, reversible doping/dedoping behavior, and inherent chemical sensitivity to acidic gases like H_2_S [14]. Previous studies have shown that these oligomers can be chemically bonded with polymer backbones to combine precise redox site control with robust film formation [15]. Among potential polymer scaffolds, poly(amic acid) (PAA) is particularly attractive due to its excellent solubility in polar organic solvents, facile chemical functionalization via its amide and carboxylic acid groups, and ability to form smooth thin films [16]. Such materials leverage the redox-active oligoaniline segments to provide intrinsic conductivity and sensitivity to acidic gases, while the polymer backbone enables robust film formation. Typically, aniline oligomers are grafted into the polymer backbone, becoming integral segments of the main chain and thus partially buried within the matrix, which can limit their direct interaction with gas analytes and slow protonation kinetics [17]. This backbone-incorporation also complicates the formation of uniform, defect-free films and may yield inconsistent sensor responses. In contrast, pendant architectures project the electroactive oligomer units outward from the polymer backbone, maximizing site exposure at the film–gas interface. Studies in graft copolymer have shown that even small variations in pendant-group structure alter functional properties compared to linear one encapsulation, underscoring the power of side-chain design to tune performance without sacrificing processability [18]. While such aniline-functionalized PAA systems have been investigated for electrochemical applications [19], to date, no prior work has reported the use of pendant aniline oligomers covalently grafted onto a PAA backbone for gas sensing—particularly H_2_S detection.

Motivated by this gap, we designed and synthesized a new class of electroactive PAA-based polymers bearing covalently attached aniline tetramer side chains. This architecture integrates the redox responsiveness and chemical sensitivity of oligoanilines with the processability of the PAA matrix. The resulting hybrid materials exhibit tunable redox behavior, and consistent conductivity profiles. As we demonstrate in this study, these features translate into high-performance H_2_S sensing behavior, with improved sensitivity, stability, and environmental durability over conventional PANI-based systems, highlighting the potential of pendant oligoaniline-functionalized polymers for next-generation gas sensing platforms.

## 2. Experimental Section

### 2.1. Chemicals and Materials

N-phenyl-1,4-phenylene diamine (99%; Sigma-Aldrich, St. Louis, MO, USA), Ammonium persulphate (APS, 97.0%; J. T. Baker, Phillipsburg, NJ, USA), N,N-Dimethyl Formamide (DMF, 99%, Merck, Darmstadt, Germany), Triethylamine (TEA, 99%, J.T. Baker), Dichloromethane (DCM, 99.5%, Macron, Crespellano, Italy), N,N-Dimethylacetamide (DMAc, 99%, Macron), Ethanol (99%), 2,6-Difluorobenzoyl chloride (DFB, 98%), Potassium carbonate (K_2_CO_3_, 99%, J.T. Baker), 3,3′,4,4′-Biphenyltetracarboxylic dianhydride (BPDA, 98%, Sigma-Aldrich), 4,4′-diaminodiphenyl ether (ODA, 97%, Sigma-Aldrich), 4-aminophenol (99%, Sigma-Aldrich), Hydrochloric acid (HCl; 37%; Riedel-de Haen, Seelze, Germany), Ammonium hydroxide solution (NH_4_OH; 28%; Riedel-de Haen, Germany), Potassium bromide (98%, KBr), Toluene (99.5%, J.T. Baker), and Sulfuric acid (H_2_SO_4_, 98%, Sigma-Aldrich) were used as received without further purification. All gases used in this study, including H_2_S, SO_2_, CO_2_, NO_2_, NH_3_, CO, and air (50 ppm; Chian Hong Gas Co., Ltd., Hong Kong, China), were used as received. Indium tin oxide glass (ITO, Ruilong Optoelectronics, Miaoli County, Taiwan) was cut into a plate measuring 25 mm × 25 mm in size and having a thickness of 0.75 mm.

### 2.2. Instruments

A laser engraver (Flux Beambox, Taipei, Taiwan) was employed to carve grooves into the ITO glass to form an ITO-based IDE that resulted in nonconductive open circuits on both sides of the glass. Mass spectra were obtained using an ion trap mass spectrometer (Ion trap MS, Daltonics Esquire 2000, Bruker, Leipzig, Germany) equipped with an Agilent electrospray ionization source. Attenuated total reflectance (ATR), a variant of FTIR spectroscopy, was measured using an FTIR spectrometer (FTIR-4100, JASCO, Oklahoma City, OK, USA; resolution, 4.0 cm^−1^; room temperature, 25 °C ± 0.5 °C) at 4000–600 cm^−1^. Electrochemical experiments were performed on Autolab (PGSTAT302N, Metrohm, Herisau, Switzerland) with a three-electrode electrochemical cell; the working, counter, and reference electrodes of this cell were a coated stainless-steel substrate, platinum plate, and saturated calomel electrode, respectively. Electrode potential was examined for up to 10 cycles between −0.2 and 1.0 V to capture the redox transitions [20] and using a 10 mV/s scan rate to provide optimal conditions to fully utilize the material’s electrochemical activity [21]. A four-point probe connected to a constant current source voltmeter system (Keithley 2400, Tektronix, Beaverton, OR, USA) was used to determine the conductivities of undoped, doped, dedoped, and redoped forms of EPAA1, 2, 3, and 5 coating.

All EPAA-coated IDE sensors were tested in-house using a bespoke gas sensing apparatus. Sensor currents were recorded with an electrometer (Keithley 2450, Keithley Instruments, Solon, OH, USA), while the analyte stream was prepared by blending compressed air with 50 ppm H_2_S from a standard gas cylinder. The resulting mixture passed through a programmable mass-flow controller (MFC; F-201CB Mass-flow Con-troller, Bronkhorst, Ruurlo, The Netherlands), which precisely regulated the flow rates of both carrier and test gases before delivering them into the mixing chamber to achieve the desired analyte concentrations.

### 2.3. Synthesis of Pendant Electroactive Diamine Monomer (PEDA)

**Scheme 1:**
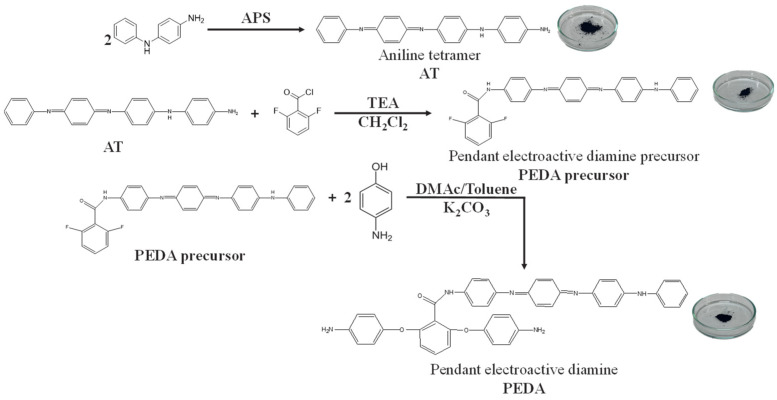
Synthetic route of PEDA.

A typical scheme for the preparation of the pendant electroactive diamine monomer is provided in Scheme 1. Firstly, a solution containing N-phenyl-1,4-phenylenediamine (3.68 g, 20 mmol) was prepared in a solvent mixture of DMF (100 mL), H_2_O (100 mL), and HNO_3_ (25 mL) stirring at 600 rpm. The emeraldine (EM) base form of aniline tetramer (AT) was synthesized by adding APS (2.28 g, 10 mmol) as the oxidant under 600 rpm stirring, dedoping with 1 mol/L NH_3_⋅H_2_O (400 mL), filtered and washed, and finally vacuum drying at 55 °C to obtain AT. Next, it was mixed 0.9161 g (0.5 mmol) AT and 1 mL TEA with 50 mL DCM, and stirred at 600 rpm for 5 min with nitrogen gas flowing throughout. A mixture of 10 mL DCM and 0.6 mL DFB was dropped in while maintaining 600 rpm stirring. Then, DCM was removed by rota-vap, and an appropriate amount of 1 M HCl was added and shaken for 1 h to precipitate. After the suction filtered, the solid was vacuum dried at 60 °C for 6 h to obtain pendant electroactive diamine (PEDA) precursor. Finally, a solution of 4-aminophenol (1.47 g, 0.6 mmol) and K_2_CO_3_ (1.04 g, 15 mmol) was prepared in 40 mL DMAc with 5 mL toluene, then refluxed under nitrogen at 110 °C, stirring at 600 rpm, for 1 h to azeotropically remove water. Separately, DFAT (0.89 g, 1.5 mmol) was dissolved in 20 mL DMAc and added dropwise to it over 10 min under 600 rpm stirring. The temperature was raised to 140 °C for 2 h to eliminate toluene, then increased to 160 °C and maintained for 18 h while maintaining 600 rpm stirring to complete the coupling reaction. After cooling to room temperature, the reaction mixture was poured into 400 mL of 1 M HCl(aq) and stirred at 600 rpm for 4 h to precipitate the product. The solid was collected by centrifugation (9000 rpm, 30 min), redispersed in 1 M NaOH(aq), centrifuged again under the same conditions, and finally freeze-dried to yield the PEDA.

### 2.4. Synthesis of Electroactive Poly(amic acid) (EPAA)

**Scheme 2:**
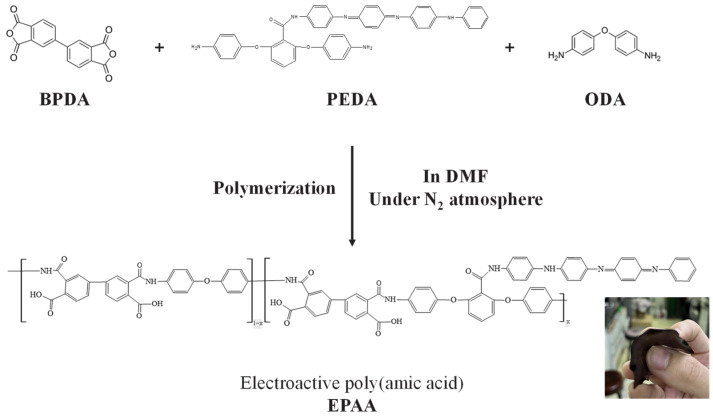
Schematic route of EPAA.

Scheme 2 depicts the synthetic pathway for the EPAA series, and Table 1 details each formulation’s composition, where EPAAX denotes that PEDA comprises X wt% of the total diamine content. For instance, EPAA3 contains 3 wt% PEDA relative to the total diamine fraction. To prepare EPAA3, a three-neck round-bottom flask equipped with a mechanical stirrer was charged with 25 g DMF, 0.99 g of ODA, and 0.08 g of PEDA. The mixture was stirred at 2000 rpm at room temperature under a continuous N_2_ purge to remove residual moisture. Once the diamines were fully dissolved, 1.47 g of BPDA was added dropwise, and the reaction proceeded under stirring at 2000 rpm for 24 h. The resulting polymer solution was then heated to 110 °C for 1 h to yield the solid EPAA3 product.

### 2.5. Preparation of EPAA Composites-Coated Sensors

**Scheme 3:**
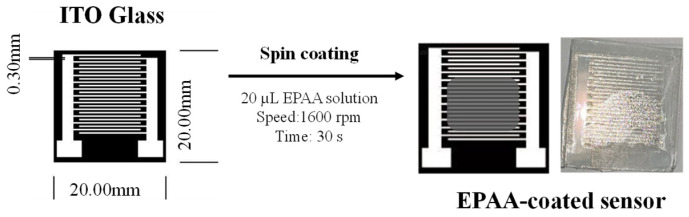
Fabrication of EPAA-coated sensors.

The spin-coating technique was used to prepare ITO coated with EPAA1, EPAA2, EPAA3, and EPAA5 [22]. A 1 wt% solution of each EPAA sample (EPAA1, EPAA2, EPAA3, and EPAA5) was first prepared in DMF under ambient stirring (Scheme 3). To fabricate the sensing layer, 20 μL of each solution was deposited onto ITO substrates and spun at 1600 rpm. The resulting films were allowed to dry overnight at room temperature, yielding uniform coatings approximately 100 ± 10 nm thick. Spin coating ensures highly controlled thin films ideal for chemiresistive measurements.

### 2.6. Gas Sensing Experiment

**Scheme 4:**
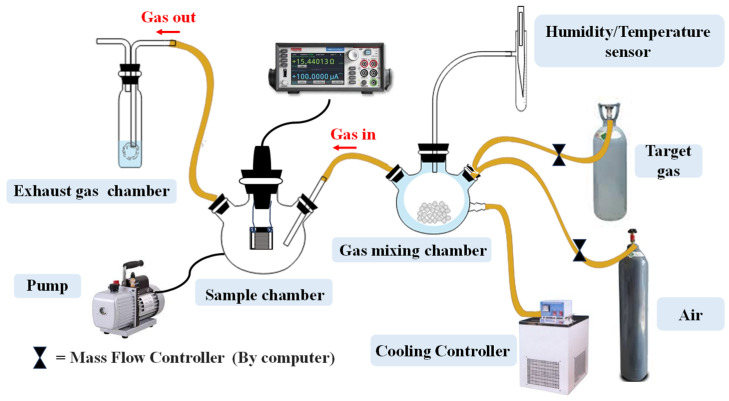
Schematic diagram of the gas sensing apparatus.

Sensor performance was assessed at room temperature (25 ± 0.5 °C) in ambient air using a custom gas-flow setup (Scheme 4). A Keithley 2450 SourceMeter supplied a constant 1 V bias across the interdigitated electrodes and recorded the transient current–time (I–t) profiles for response normalization [23,24,25]. The sensor chamber ports were plumbed to a Bronkhorst F-201CB mass-flow controller that blended dry air with certified gas standards (H_2_S, SO_2_, NO_2_, NH_3_, CO; Chiao Tai Gas Co., Ltd., Taoyuan City, Taiwan) at a total flow of 1000 sccm. Humidity dependence was studied by controlling the chamber’s water-bath temperature, holding each relative humidity (RH) condition for at least 15 min (monitored by a hygrometer) before measurement [26]. Unless otherwise noted, RH was maintained at ~60%. For comparative tests, 10 ppm H_2_S was chosen in line with OSHA’s permissible exposure limit [27]. Sensor response was normalized as [28]:

Response (%) = (I_g_ − I_a_)/I_a_ × 100 (1)

where I_a_ and I_g_ are the baseline and gas-exposed currents, respectively.

The limit of detection (LOD) was calculated as [29]:

LOD = 3σ/S(2)

with σ representing the standard deviation and S the slope of the calibration curve.

Response time was defined as the interval to reach 90% of the peak signal after gas exposure, and recovery time as the duration to return to 10% of that peak once switched to gas off [9].

## 3. Results and Discussion

### 3.1. Mass Spectrum of Electroactive Monomers (AT and PEDA)

**Figure 1:**
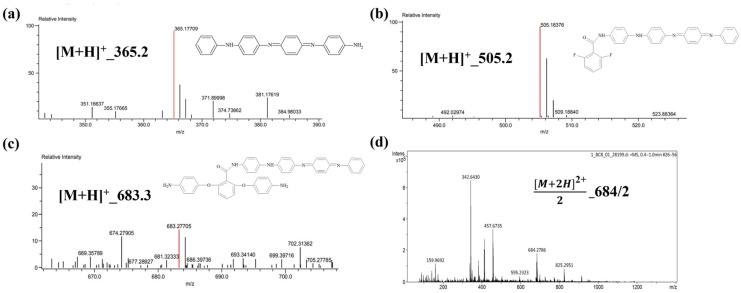
Mass spectrum of electroactive monomers (**a**) AT, (**b**) PEDA precursor, (**c**,**d**) PEDA.

In Figure 1a–c, AT, PEDA precusor, and PEDA of the molecular weights are 364.17 g/mol, 504.18 g/mol, and 682.27 g/mol, respectively. After analysis using positive ion mode mass spectra, signals with the mass-to-charge ratio (*m*/*z*) values of 365.2 (M + H)^+^, 505.2 (M + H)^+^, and 683.3 (M + H)^+^ were obtained, respectively. The signal of PEDA is not outstanding, as shown in Figure 1d. This is due to its large molecular size and primary amines at both the front and the end, which caused the protonation. The double proton adduct ion is more readily detected than the single proton, resulting in a stronger signal at [M + 2H]^2+^ [30].

### 3.2. FTIR Analyses of Electroactive Monomers (AT and PEDA)

**Figure 2:**
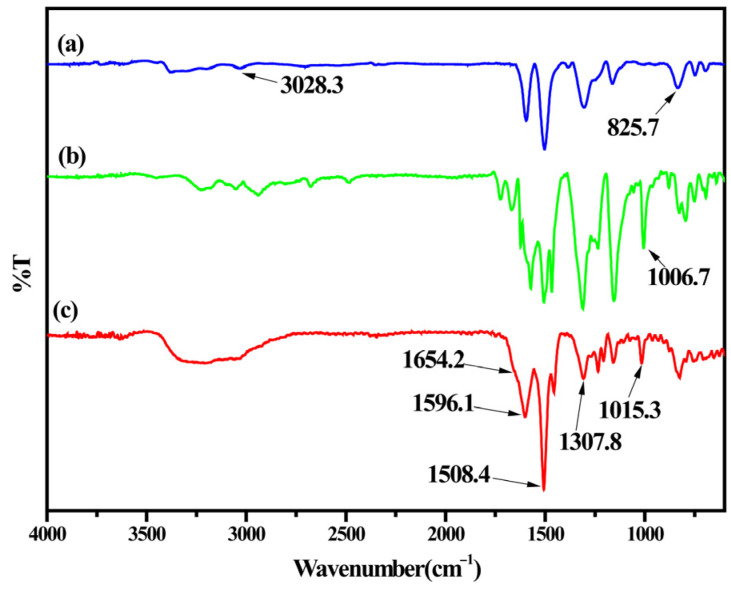
FT-IR spectra of electroactive monomers (**a**) AT, (**b**) PEDA precursor, (**c**) PEDA.

The FT-IR spectra of the electroactive monomer were verified in Figure 2. In Figure 2a, AT was verified through the characteristic functional groups, where the two peaks at 3028.3 and 825.7 cm^−1^ are attributed to the out-of-plane vibration of hydrogen atoms on the benzene ring. The absorption peak of the C–N bond in the amine group was observed at 1307.8 cm^−1^. Additionally, two peaks at 1508.4 and 1596.1 cm^−1^ corresponded to the benzene (N=B=N) rings and the quinoid (N=Q=N) in C=C, respectively. The stretching absorption peak of the N-H bond was observed at 3290.6 cm^−1^ [31]. In Figure 2b, PEDA precursor was verified through the characteristic functional groups. In addition to retaining the functional group signals of AT, PEDA precursor exhibited characteristic peaks resulting from chemical substitution. The C–F of the stretching signal was observed at 1006.7 cm^−1^, and the C=O of the acyl chloride group of the characteristic absorption peak was observed at 1654.2 cm^−1^. Finally, in Figure 2c, PEDA was verified through the characteristic functional groups. Through further chemical substitution reactions, the C–F stretching signal of precursor disappears, while the C–O bond of the ether group gives rise to the characteristic absorption peak at a similar position observed at 1015.3 cm^−1^. Based on the functional group spectra obtained from FT-IR spectra, the initial results indicated the successful synthesis in this experiment of AT and PEDA.

### 3.3. ATR-FTIR Analyses of EPAA

**Figure 3:**
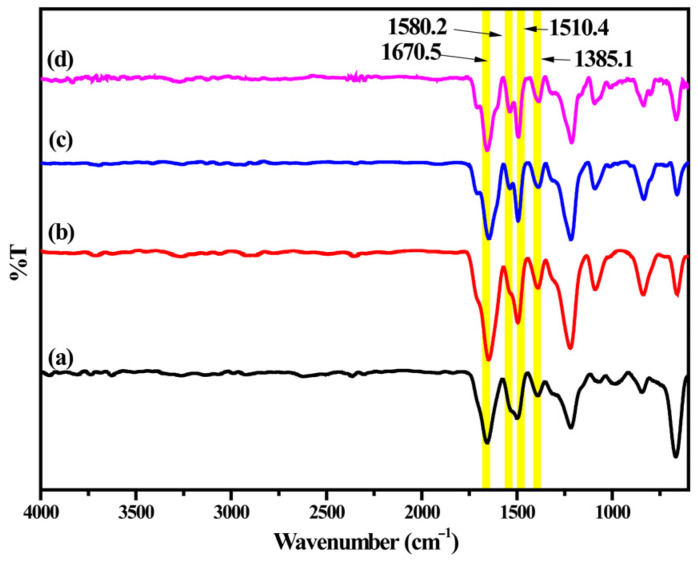
ATR-FTIR spectra of (**a**) EPAA1, (**b**) EPAA2, (**c**) EPAA3, and (**d**) EPAA5.

The successful incorporation of PEDA into the EPAA backbone is clearly evidenced by ATR-FTIR (Figure 3), which reveals loading-dependent variations in several diagnostic vibrational bands. All EPAA films—regardless of tetramer content—exhibit two prominent peaks at 1580.2 cm^−1^ and 1510.4 cm^−1^, attributable to the quinoid and benzenoid C=C stretches of the aniline tetramer, respectively. These wavenumbers closely align with values reported for other grafted oligoaniline systems, confirming the chemical identity of the pendant groups [32]. In addition, the presence of the amide C=O stretching at 1670.5 cm^−1^ and C–N bending at 1385.1 cm^−1^ across EPAA1-EPAA5 verifies the formation of the poly(amic acid) backbone structure. In conducting-polyaniline-related systems, the intensity ratio of the quinoid to benzenoid C=C stretches (Q/B) provides a semi-quantitative measure of the polymer’s oxidation level [33]. Calculated from the quinoid peak and the benzenoid peak, the Q/B ratio is highest for EPAA1, reflecting the pronounced oxidation signature of well-isolated aniline tetramer pendants at low loading. As the pendant content increases to EPAA2 and EPAA3, the Q/B ratio decreases modestly, indicating that closer proximity of tetramer units induces electronic interactions that slightly moderate the apparent oxidation state. At the highest loading (EPAA5), the Q/B ratio reaches its lowest value, suggesting that excessive pendant density leads to intra-chain interactions or early aggregation. This trend demonstrates that the redox behavior of EPAA films can be tuned by adjusting tetramer side-chain content.

### 3.4. Electrochemical Redox Properties of EPAA

**Figure 4:**
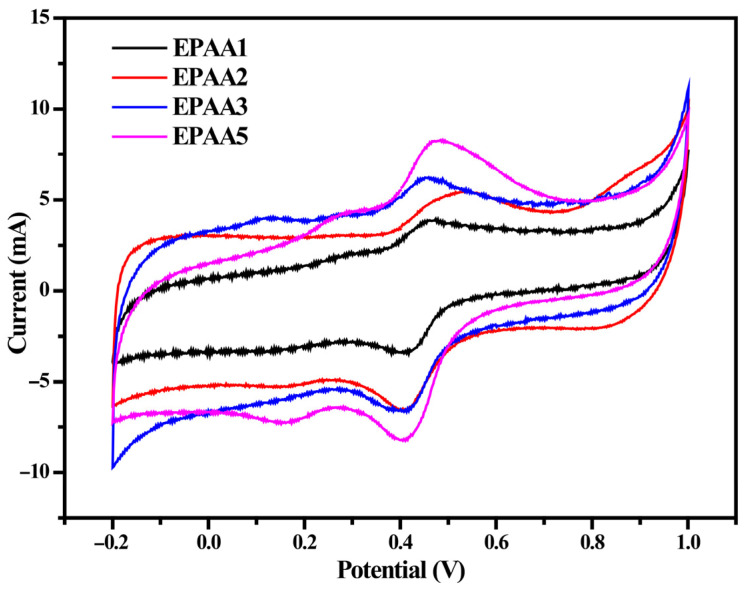
Cyclic voltammogram showing the redox capabilities of EPAA1, EPAA2, EPAA3, and EPAA5.

CV performance was carried out in 1.0 M H_2_SO_4_ over the potential window of −0.2 V to 1.0 V at various scan rates, and 5 mV s^−1^ was chosen for direct comparison in Figure 4 [34]. Each EPAA film (EPAA1, EPAA2, EPAA3, EPAA5) exhibited two distinct redox couples that reflect stepwise oxidation of the aniline tetramer side chains. Initially, all nitrogen centers reside in the fully reduced leucoemeraldine form (amine-only nitrogens). The first oxidation event converts half of these amine groups to imines, yielding the emeraldine state, and a second oxidation transforms the remaining amines into imines, producing the pernigraniline state [35]. The peak currents for EPAA1 were +2.19 mA/−3.46 mA and +3.96 mA/−3.45 mA; for EPAA2, +3.02 mA/−6.58 mA and +5.47 mA/−5.32 mA; for EPAA3, +4.05 mA/−6.66 mA and +6.24 mA/−6.16 mA; and for EPAA5, +4.64 mA/−8.25 mA and +8.29 mA/−7.32 mA. As the PEDA content increases, the oxidation currents rise accordingly: EPAA2, EPAA3, and EPAA5 exhibit 1.55-, 1.84-, and 2.25-fold greater redox activity than EPAA1, respectively. The results demonstrate that grafting PEDA onto the poly(amic acid) backbone significantly enhances its redox capability. This clear, sequential redox behavior confirms the accessibility and reversibility of the pendant electroactive groups across the series. The oxidation potential gradually shifts to more positive values with higher PEDA content. As the density of tetramer pendants grows, π–π stacking and closer packing of redox sites stabilize the reduced state, raising the energy barrier for oxidation and thus shifting the peak to more positive potentials. Dense pendant loading increases the film’s pseudocapacitance, associated with fast redox processes, which can distort voltammograms and shift peak potentials [36]. In EPAA films, higher side-chain content amplifies these capacitive effects, further driving the apparent oxidation potentials upward.

### 3.5. Conductivity Test of EPAA by Four-Probe

A four-probe setup was employed to evaluate the baseline electrical conductivities of the EPAA thin-film samples containing varying amounts of PEDA. After recording these initial values, each film was exposed to hydrogen sulfide gas to serve as a dopant, and the resulting increase in conductivity was measured. The films were then dried to remove the H_2_S (dedoping), and conductivity readings were taken once more. Finally, a second H_2_S exposure (redoping) was performed and the conductivity was measured for a fourth time, allowing us to assess both the reversibility and repeatability of the doping process (Table 2). Under undoped conditions, the conductivities of EPAA1, EPAA2, EPAA3, and EPAA5 were 2.19 × 10^−6^, 4.62 × 10^−6^, 7.53 × 10^−6^, and 2.01 × 10^−6^ S/cm, respectively, with EPAA3 showing the highest intrinsic conductivity. Introduction of H_2_S boosted the conductivity of all samples by roughly fiftyfold, demonstrating the strong p-type doping effect of sulfur species. Once the films were dried and the H_2_S removed, their conductivities returned nearly to the original baseline levels, confirming that the doping was largely reversible. A subsequent H_2_S exposure again elevated conductivities to comparable values, illustrating not only the material’s robust doping capacity but also its potential for repeated gas sensing cycles without permanent degradation.

### 3.6. Morphology of EPAA Films

**Figure 5:**
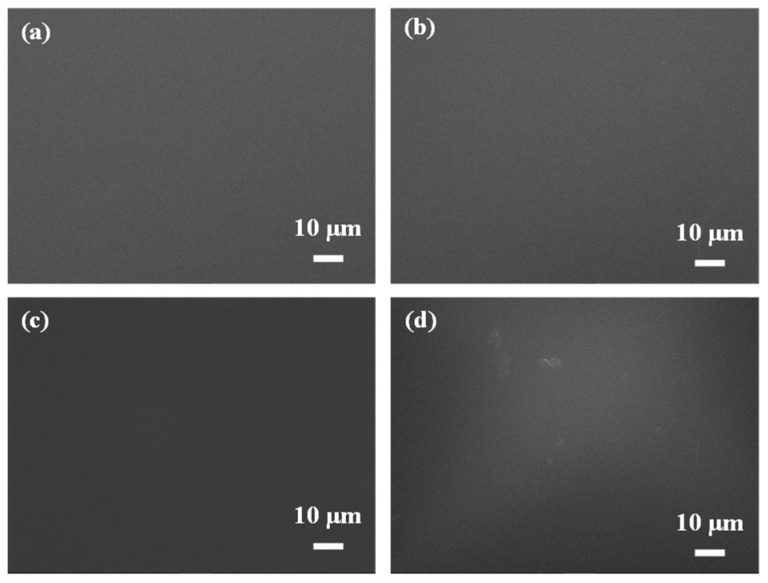
SEM images of (**a**) EPAA1, (**b**) EPAA2, (**c**) EPAA3, and (**d**) EPAA5.

The surface morphology of EPAA films with varying aniline-tetramer (PEDA) content was characterized by SEM (Figure 5). Films containing 1–3 wt% PEDA (EPAA1–EPAA3) exhibit continuous, defect-free surfaces (Figure 5a–c). Their topographies appear smooth and uniform, with no discernible phase separation or void formation. The even contrast and absence of particulate features suggest that, up to 3 wt% loading, the pendant tetramer units are well dispersed within the poly(amic acid) matrix. In contrast, the EPAA5 sample (5 wt% PEDA) shows the emergence of discrete microscale aggregates (Figure 5d). These bright, clustered domains—absent in lower-loading films—likely result from tetramer–tetramer π–π stacking that exceeds the solubility threshold of the polymer backbone. The aggregates range from a few micrometers to tens of micrometers in diameter and are distributed irregularly across the film surface.

### 3.7. Gas Sensing Performance

#### 3.7.1. Sensitivity and Calibration Curve

**Figure 6:**
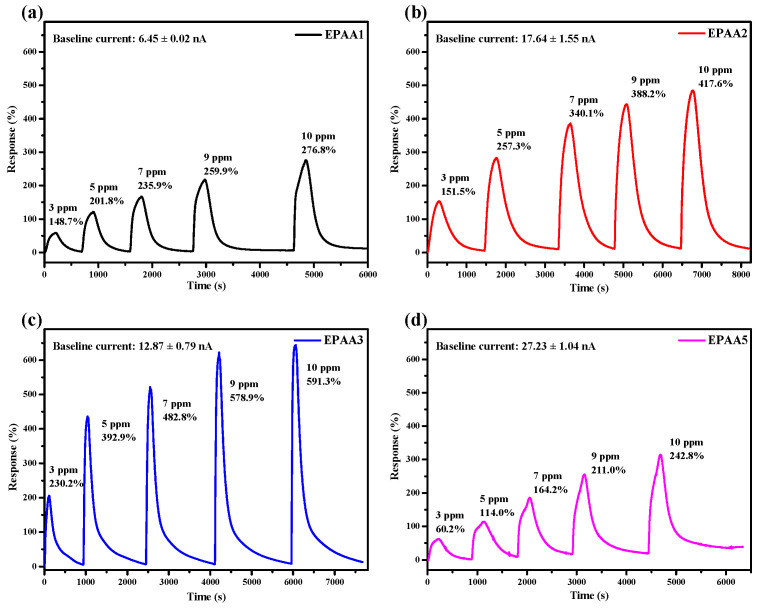
Sensitivity of the IDE sensor device with the (**a**) EPAA1, (**b**) EPAA2, (**c**) EPAA3, and (**d**) EPAA5 coating at various concentrations of H_2_S.

The sensitivity of the EPAA-based IDE sensors toward H_2_S gas was evaluated across a concentration range of 3 to 10 ppm, as shown in Figure 6. The baseline electrical currents of the IDE sensors coated with EPAA1, EPAA2, EPAA3, and EPAA5 were 6.45 ± 0.02 nA, 17.64 ± 1.55 nA, 12.87 ± 0.79 nA, and 27.23 ± 1.04 nA, respectively. Among them, EPAA3 displayed the highest sensitivity across all tested concentrations, reflecting its superior proton doping capacity. Specifically, the response values of EPAA3 to 3, 5, 7, 9, and 10 ppm H_2_S were 230.2%, 392.9%, 482.8%, 578.9%, and 591.3%, respectively. Comparatively, EPAA2 demonstrated slightly lower responses of 151.5%, 257.3%, 340.1%, 388.2%, and 417.6%, while EPAA1 showed more moderate responses of 148.7%, 201.8%, 235.9%, 259.9%, and 276.8%. Although EPAA5 contains the highest loading of PEDA (5 wt%), its responses—60.2%, 114.0%, 164.2%, 211.0%, and 242.8%—were markedly lower. This inverted trend at 5 wt% indicates a loading limit for pendant electroactive diamine: beyond ~3 wt%, excessive PEDA pendants aggregate and sterically hinder uniform proton access and electron percolation along the backbone. In EPAA5, these aggregated domains both block efficient formation of conductive pathways and disrupt backbone conjugation, resulting in reduced H_2_S-induced doping efficiency and a lower overall sensor response. The enhanced sensitivity of EPAA3 is attributed to its optimal loading of PEDA, which introduces redox-active sites along the polymer chain. These pendant groups facilitate efficient protonation by H^+^ ions derived from H_2_S dissociation in humid conditions, thereby boosting the overall charge transport and amplifying the sensor signal.

**Figure 7:**
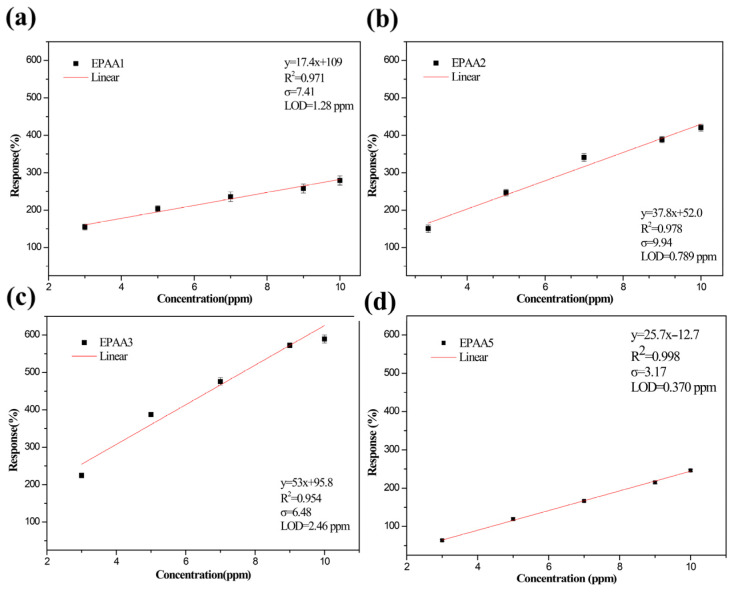
Linear calibration curves of (**a**) EPAA1, (**b**) EPAA2, (**c**) EPAA3, and (**d**) EPAA5 IDE sensors.

Figure 7 presents the calibration plots of sensor response versus H_2_S concentration for IDEs coated with EPAA1, EPAA2, EPAA3, and EPAA5. Over the 3–10 ppm range, each set of data exhibits a strong linear relationship: EPAA1 follows y = 17.4·x + 109.0 (R^2^ = 0.971), EPAA2 follows y = 37.8·x + 52.0 (R^2^ = 0.978), EPAA3 follows y = 53.0·x + 95.8 (R^2^ = 0.954), and EPAA5 follows y = 25.7·x + 12.7 (R^2^ = 0.997). These high correlation coefficients confirm excellent proportionality between gas concentration and current change, with EPAA3 delivering the steepest slope—indicating the greatest sensitivity per ppm—and EPAA5 exhibiting the most precise fit. The high R^2^ values (>0.95) confirm excellent linearity across the tested concentration window, with EPAA5 showing the best fitting precision and EPAA3 the strongest concentration-dependent sensitivity. From the slope and the standard deviation (σ), the theoretical limits of detection were estimated as 1.28 ppm for EPAA1, 0.79 ppm for EPAA2, 2.46 ppm for EPAA3, and 0.37 ppm for EPAA5 using Equation (2). In practice, slight increases in the measured LOD may occur due to instrumental and operational variability, but these values nonetheless highlight the superior detectability of the PEDA-modified films—within the low-ppm regime.

#### 3.7.2. Response and Recovery Time, Selectivity, and Repeatability

**Figure 8:**
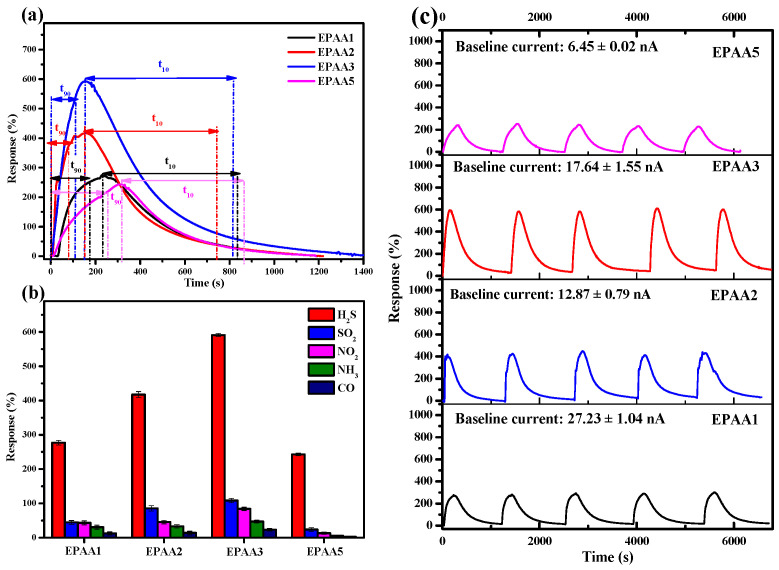
(**a**) Comparison response of EPAA1-, EPAA2-, EPAA3-, and EPAA5-coated IDE sensors exposed to 10 ppm H_2_S. (**b**) Selectivity of the aforementioned sensors exposed to five different gases (concentration = 10 ppm). (**c**) Repeatability of the aforementioned IDE sensors at an H_2_S concentration of 10 ppm.

The gas sensing performance of the EPAA films was evaluated by measuring their response and recovery behavior upon exposure to 10 ppm H_2_S (Figure 8a). The response time varies noticeably with PEDA loading. EPAA3 exhibited the fastest response at 108 s, while EPAA1, EPAA2, and EPAA5 required 165 s, 215 s, and 254 s, respectively. The superior response speed of EPAA3 suggests that 3 wt% PEDA represents an optimal grafting density, where sufficient active redox sites are uniformly distributed, enabling rapid protonation and charge transfer during H_2_S exposure. Conversely, the recovery times show an inverse trend. EPAA5 demonstrated the fastest recovery at 787 s, followed by EPAA1 (890 s), while EPAA2 and EPAA3 exhibited prolonged recovery of 1150 s and 1128 s, respectively. This behavior can be attributed to the interaction strength between H_2_S and the electroactive sites. Moderate PEDA loading (EPAA2 and EPAA3) results in a higher density of accessible active sites that engage strongly with H_2_S, enhancing sensitivity but slowing desorption. In contrast, excessive PEDA in EPAA5 likely leads to pendant aggregation and reduced binding efficiency, facilitating quicker H_2_S desorption and recovery.

To evaluate the selectivity of the as-fabricated sensors, four common toxic and hazardous gases beyond H_2_S were also tested. Figure 8b compares the responses of EPAA1, EPAA2, EPAA3, and EPAA5 to 10 ppm of H_2_S, SO_2_, NO_2_, NH_3_, and CO. EPAA3 (3 wt% PEDA) again demonstrated outstanding selectivity toward 10 ppm H_2_S, generating a 591.3% response—more than five times its reaction to SO_2_ (108.1%) and nearly seven times its response to NO_2_ (84.2%). It also outperformed NH_3_ and CO, which induced only 47.3% and 23.4% responses, respectively. EPAA2 followed a similar trend (417.6% for H_2_S vs. 85.1%, 45.5%, 33.4%, and 14.6% for SO_2_, NO_2_, NH_3_, and CO), while EPAA1 and EPAA5 showed lower absolute responses but still maintained at least an order-of-magnitude preference for H_2_S over other gases. This pronounced discrimination stems from the optimal density of aniline-tetramer pendants in EPAA3, which creates a conjugated network uniquely tuned to interact with sulfide species. At 5 wt% PEDA (EPAA5), the excess pendants begin to disrupt that network, reducing H_2_S sensitivity (242.8%) and narrowing the gap between H_2_S and other gases responses. Furthermore, we evaluated EPAA3 in a simple mixed-gas scenario (10 ppm H_2_S + 10 ppm SO_2_). As shown in Supplementary Figure S2, the combined atmosphere produced a response of 678.1%, compared to 591.3% for pure H_2_S, indicating that SO_2_ co-presence slightly amplifies the overall signal but does not compromise the sensor’s primary H_2_S detection capability.

To evaluate the reproducibility of the EPAA-based sensors, repeatability tests were performed by subjecting each film (EPAA1, EPAA2, EPAA3, and EPAA5) to five consecutive exposure–recovery cycles with 10 ppm H_2_S gas (Figure 8c). Across all samples, the sensors demonstrated consistent response magnitudes with no significant signal degradation or baseline drift throughout repeated use. The average response values were recorded as 287.1 ± 9.8% for EPAA1, 424.1 ± 12.6% for EPAA2, 589.1 ± 7.4% for EPAA3, and 239.5 ± 10.8% for EPAA5. Among them, EPAA3 exhibited not only the highest sensitivity but also the lowest standard deviation, highlighting its excellent reproducibility under repeated H_2_S exposure. The minimal variability in response cycles for EPAA3 reflects the optimal pendant loading of 3 wt%, which provides a uniform distribution of accessible redox-active sites without the aggregation or structural heterogeneity observed at higher loading (EPAA5). In contrast, the larger fluctuations seen for EPAA5 may result from excess pendant content leading to localized aggregation and less uniform film morphology, which can compromise sensor consistency. Overall, these results confirm that the EPAA system, particularly at optimized loading, delivers reliable, repeatable performance, an essential requirement for practical sensing applications in industrial or environmental monitoring.

#### 3.7.3. Stability and the Effect of Humidity

**Figure 9:**
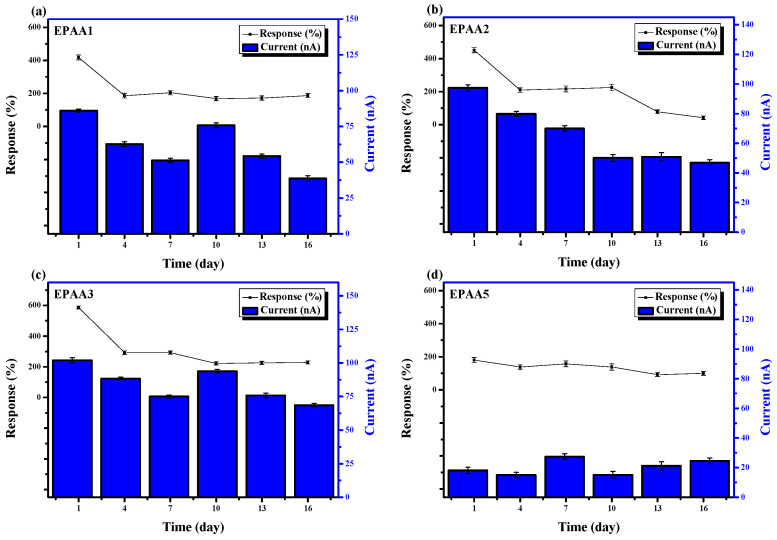
Stability of the (**a**) EPAA1, (**b**) EPAA2, (**c**) EPAA3, and (**d**) EPAA5-coated sensors (concentration = 10 ppm).

Over a 16-day period under ambient storage, all four EPAA-coated IDE sensors were challenged daily with 10 ppm H_2_S to evaluate their environmental stability (Figure 9). Aside from an unusually high initial response on Day 1, each formulation retained more than 80% of its 16-day peak sensitivity, demonstrating minimal performance drift. In particular, the EPAA3 sensor continued to deliver the strongest signal after 16 days, underscoring its exceptional combination of high sensitivity and long-term durability. These findings verify that our PEDA-grafted poly(amic acid) films sustain both their electroactive behavior and gas sensing capability in practical conditions, supporting their promise for reliable, real-world H_2_S monitoring.

**Figure 10:**
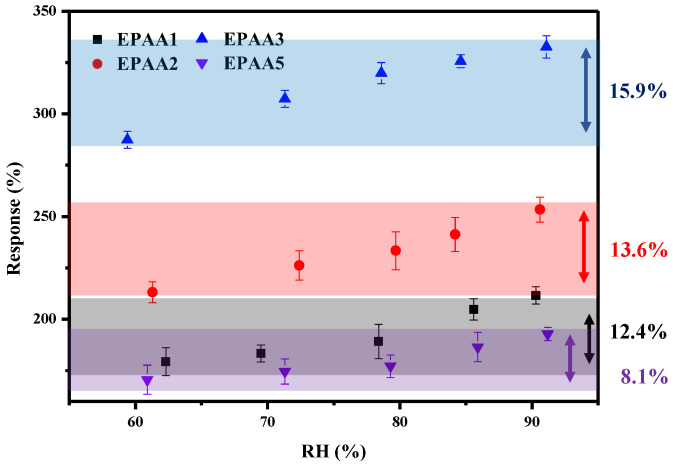
The effect of relative humidity on the response of EPAA1, EPAA2, EPAA3, and EPAA5 sensor.

Humidity exerts a complex influence on chemiresistive sensors by modulating water adsorption, proton availability, reaction kinetics at the polymer interface, and—even at extreme levels—electrode stability [37]. In our EPAA films, water molecules absorbed into the polymer matrix facilitate H_2_S dissociation into H^+^ and HS^−^, enhancing doping of aniline units. As shown in Figure 10, when relative humidity (RH) was varied from 60% to 90%, the responses to 10 ppm H_2_S exhibited standard deviations of 12.4% for EPAA1, 13.6% for EPAA2, 15.9% for EPAA3, and 8.1% for EPAA5. These values reflect moderate humidity dependence, indicating that fluctuations in ambient moisture could lead to unreliable readings in real-world conditions. Moreover, higher RH levels also prolong the desorption of both water and sulfide species, extending recovery times. Since the variability under elevated humidity was acceptable yet could compromise cycle speed, we standardized all subsequent measurements at 60% RH to strike a balance between sensitivity, stability, and response kinetics. Looking ahead, improving moisture resistance will be a critical area of development. Strategies such as integrating hydrophobic domains or optimizing the distribution of pendant electroactive diamine groups to limit excessive water uptake could yield humidity-invariant EPAA variants that maintain robust proton-driven sensing without sacrificing recovery speed or long-term stability.

Table 3 compares key performance metrics of various conductive-polymer-based H_2_S sensors. Notably, the EPAA3-coated sensor achieves a response of 591% at 10 ppm H_2_S at room temperature, with a response time of 108 s and a recovery time of 1128 s. While the rapid response highlights the effectiveness of the side-chain architecture for proton-doping, the extended recovery arises from the deep protonation of the densely grafted tetramer side chains: while the high pendant density maximizes charge–carrier injection (and thus response value), it also retards desorption of H_2_S. To mitigate such prolonged recovery times, as seen with the EPAA3 film’s retarded H_2_S desorption due to deep protonation, efforts in gas sensor design focus on optimizing operational conditions and material properties. Critically, the design of heterostructure composite materials significantly improves recovery rates. These materials enhance gas desorption by improving catalytic activity, forming electron depletion layers, increasing adsorption sites, and tuning band structures Despite its extended recovery, **EPAA3’s** combination of ultra-high sensitivity, excellent selectivity, robust repeatability (±<7% drift over five cycles), and stability (>80% retention over 16 days) makes it a standout material for reliable H_2_S monitoring in environmental and industrial applications.

### 3.8. Gas Sensing Mechanism

**Figure 11:**
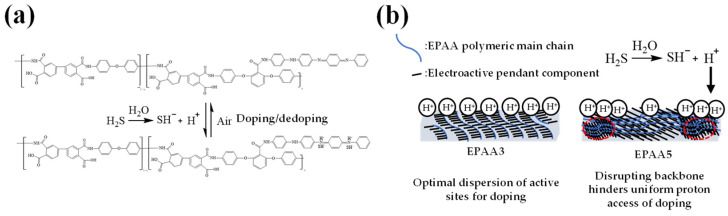
(**a**) Gas sensing mechanism of EPAA doping and dedoping; (**b**) graphical illustration of gas sensing for EPAA3 and EPAA5.

When H_2_S enters the humidified sensing chamber, it dissolves in the adsorbed water layer and dissociates into H^+^ and HS^−^ ions [41]. As shown in Figure 11a, these protons then protonate the aniline units along the poly(amic acid) backbone, converting neutral benzenoid rings into positively charged emeraldine and pernigraniline species and thereby injecting mobile charge carriers that increase the material’s conductivity. In EPAA films, grafting pendant electroactive diamine (PEDA) groups amplifies this effect: each pendant aniline tetramer brings additional redox-active sites that are spatially decoupled from the main chain, promoting more efficient protonation and faster electron hopping along the conjugated network. This side-chain architecture both raises the density of protonation sites and improves the dispersion of active domains, yielding larger current changes upon H_2_S exposure. However, there is a practical limit to how much PEDA can be incorporated (Figure 11b). SEM analysis (Figure 5) confirms that EPAA1–EPAA3 (1–3 wt% PEDA) form smooth, defect-free films with uniformly dispersed tetramer units, ensuring maximal exposure of redox sites. However, at 5 wt% loading (EPAA5), distinct microscale aggregates emerge, as excessive π–π stacking of tetramer pendants exceeds the matrix’s solubility threshold. These aggregates sterically hinder proton diffusion and break conductive pathways, causing diminished redox activity and slower response. As a result, EPAA3 strikes the optimal balance of uniform morphology with high pendant density to yield the best sensitivity, kinetics, and reproducibility.

## 4. Conclusions

In this work, electroactive poly(amic acid) (EPAA) films grafted with varying loadings of aniline tetramer pendants (PEDA) were successfully synthesized and characterized. SEM revealed smooth, crack-free morphologies for EPAA1–EPAA3 and the onset of tetramer aggregation in EPAA5. CV showed two well-defined redox couples whose peak currents increased with PEDA content, evidence of the successful grafting of the pendant groups and strongly enhanced electroactivity. Four-probe conductivity measurements further demonstrated the electrical tunability of the films, establishing a structure–property relationship that underpins the observed gas sensing behavior. Among the formulations, EPAA3 (3 wt% PEDA) struck the best balance: at 10 ppm H_2_S it delivered a 591% signal change in just 108 s, far exceeding its responses to SO_2_, NO_2_, NH_3_, and CO, and showed excellent linearity with a low detection limit. Repeatability tests (five cycles) yielded only ~7.4% relative variation, and 16-day ambient stability retained over 80% of its Day 2–16 response—outperforming as-prepared samples in long-term durability. Although its recovery time (1128 s) reflects deep protonation of dense side chains, this trade-off underscores the high sensitivity achieved. Future work will focus on film thinning, micro/nanostructuring, or heterostructure composites to accelerate desorption kinetics and achieve near real-time recovery. Overall, EPAA3 demonstrates a promising balancing of sensitivity, selectivity, processability, and stability, laying the groundwork for next-generation, scalable H_2_S sensors in environmental and industrial applications.

## Figures and Tables

**Table 1:** Compositions of EPAA1, EPAA2, EPAA3, and EPAA5.

Code	Dianhydride	Diamine *	
	BPDA Content	ODA Content	PEDA Content
EPAA1	1.47 g	0.99	0.03
EPAA2		0.98	0.05
EPAA3		0.97	0.08
EPAA5		0.96	0.13

* The molar ratio of diamines were equal to the molar ratio of dianhydride.

**Table 2:** Conductivity comparison of EPAA1, EPAA2, EPAA3, and EPAA5 in intrinsic, doped, dedoped and redoped forms.

Code	Conductivity (S/cm)			
	Intrinsic	Doped	Dedoped	Redoped
EPAA1	2.19 × 10^−6^	1.69 × 10^−4^	1.16 × 10^−6^	9.59 × 10^−5^
EPAA2	4.62 × 10^−6^	1.97 × 10^−4^	1.32 × 10^−6^	9.62 × 10^−5^
EPAA3	7.53 × 10^−6^	2.51 × 10^−4^	1.45 × 10^−6^	9.56 × 10^−5^
EPAA5	2.01 × 10^−6^	1.82 × 10^−4^	1.02 × 10^−6^	9.46 × 10^−5^

**Table 3:** Performance comparison of other conductive polymer-based H_2_S sensors with various formulation with present work.

Sample Code	Response	Gas Concentration (ppm)	Response/Recovery Time (s)	Ref.
PANI–PEO	25%	10	120/250	[10]
SnO_2_/rGO/PANI	91.11%	10	80/88	[11]
PANI/CuO	188%	25	306/296	[38]
PANI/WO_3_/CuO	90.1%	10	353/4958	[39]
CS/1,3-propanediol conductive polymer gel	32%	10	23/17	[40]
EPAA3	591.3%	10	108/1128	This work

## Data Availability

The data that support the findings of this study are available from the corresponding author, but restrictions apply to the availability of these data, which were used under license for the current study, and so are not publicly available. The data are, however, available from the authors upon reasonable request and with permission of the funding party, Ministry of Science and Technology, Taiwan, R.O.C.

## Supplementary Materials

The following supporting information can be downloaded at: https://www.mdpi.com/article/10.3390/polym17141915/s1, Figure S1: Sensing responses EPAA3 sensors (at the working temperature) exposed to H2S at a concentration of 10 ppm; Figure S2: Response of EPAA3 to 10 ppm H_2_S alone and to gas mixtures containing 10 ppm H_2_S plus 10 ppm SO_2_.
