# Supplementary material for: Electroactive Poly(amic acid) Films Grafted with Pendant Aniline Tetramer for Hydrogen Sulfide Gas Sensing Applications
Source: Polymers (Basel). 2025 Jul 11;17(14):1915. doi: 10.3390/polym17141915 (PMC12299664; doi:10.3390/polym17141915)
Supplement: Supplementary file 1 [file polymers-17-01915-s001.zip › EPAA_gas_sensing_polymers_Supplementary information.pdf]

Supplementary information

Electroactive Poly(amic acid) Films Grafted with Pendant Aniline Tetramer for Hydrogen Sulfide Gas Sensing Applications

Kun-Hao Luo, Yun-Ting Chen, Hsuan-Yu Wu, Zong-Kai Ni and Jui-Ming Yeh*

Department of Chemistry, Chung Yuan Christian University, Chung Li District, Taoyuan City, 32023, Taiwan, ROC

Corresponding author:

Prof. Jui-Ming Yeh

E-mail address: juiming@cycu.edu.tw

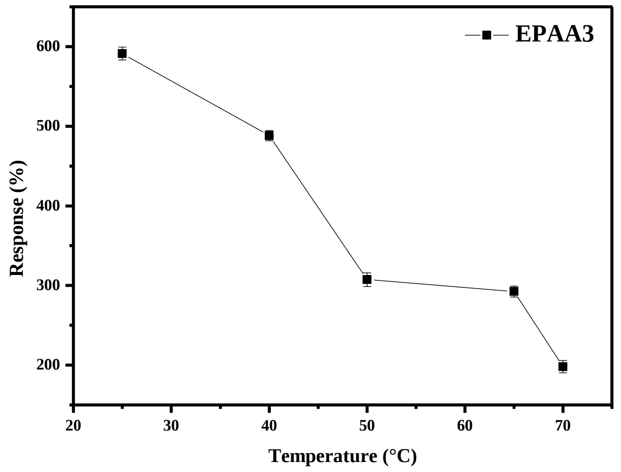

Figure S1. Sensing responses EPAA3 sensors (at the working temperature) exposed to H_2S at a concentration of 10 ppm.

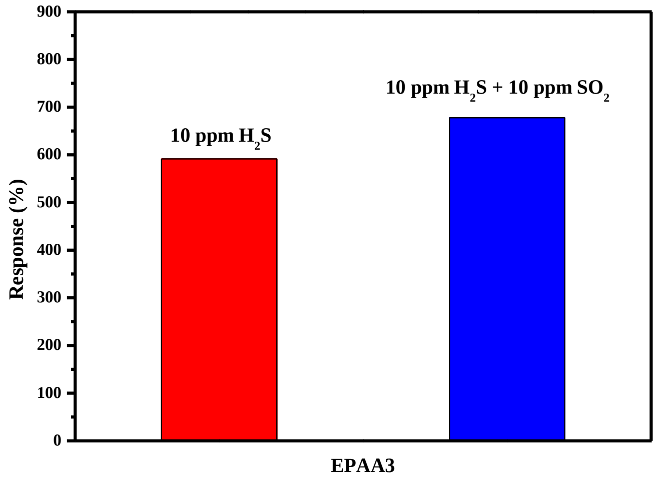

Figure S2. Response of EPAA3 to 10 ppm H_2S alone and to gas mixtures containing 10 ppm H_2S plus 10 ppm SO_2.
